# Compound phenotype in a girl with r(22), concomitant microdeletion 22q13.32-q13.33 and mosaic monosomy 22

**DOI:** 10.1186/s13039-018-0375-3

**Published:** 2018-04-27

**Authors:** Anna A. Kashevarova, Elena O. Belyaeva, Aleksandr M. Nikonov, Olga V. Plotnikova, Nikolay A. Skryabin, Tatyana V. Nikitina, Stanislav A. Vasilyev, Yulia S. Yakovleva, Nadezda P. Babushkina, Ekaterina N. Tolmacheva, Mariya E. Lopatkina, Renata R. Savchenko, Lyudmila P. Nazarenko, Igor N. Lebedev

**Affiliations:** 1 Research Institute of Medical Genetics, Tomsk NRMC, Tomsk, Russia; 2 Diagnostic Center of the Altai Region, Barnaul, Russia; 3 Siberian State Medical University, Tomsk, Russia

**Keywords:** Compound phenotype, Phelan-McDermid syndrome, *FAM19A5* gene, Ring chromosome 22, Chromosome 22 monosomy

## Abstract

**Background:**

Ring chromosome instability may influence a patient’s phenotype and challenge its interpretation.

**Results:**

Here, we report a 4-year-old girl with a compound phenotype. Cytogenetic analysis revealed her karyotype to be 46,XX,r(22). aCGH identified a 180 kb 22q13.32 duplication, a *de novo* 2.024 Mb subtelomeric 22q13.32-q13.33 deletion, which is associated with Phelan-McDermid syndrome, and a maternal single gene 382-kb *TUSC7* deletion of uncertain clinical significance located in the region of the 3q13.31 deletion syndrome. All chromosomal aberrations were confirmed by real-time PCR in lymphocytes and detected in skin fibroblasts. The deletions were also found in the buccal epithelium. According to FISH analysis, 8% and 24% of the patient’s lymphocytes and skin fibroblasts, respectively, had monosomy 22.

**Conclusions:**

We believe that a combination of 22q13.32-q13.33 deletion and monosomy 22 in a portion of cells can better define the clinical phenotype of the patient. Importantly, the *in vivo* presence of monosomic cells indicates ring chromosome instability, which may favor karyotype correction that is significant for the development of chromosomal therapy protocols.

**Electronic supplementary material:**

The online version of this article (10.1186/s13039-018-0375-3) contains supplementary material, which is available to authorized users.

## Background

Terminal deletions at 22q13 are often associated with ring chromosome formation. To date, no more than a hundred patients with r(22) have been described, and their clinical phenotype is similar to those with a terminal 22q deletion [1–7]. Ring chromosomes are known to be unstable during mitotic divisions: the ring may change in size or may be lost, and dicentric and interlocking rings may appear [8, 9]. The loss of the ring chromosome followed by the amplification of the remaining normal homolog in induced pluripotent stem cells (iPSCs) formed the basis of *in vitro* karyotype correction and chromosomal therapy [10–12].

A combination of r(22) and cells with monosomy for chromosome 22 was described previously in one of two mentally retarded monozygotic twins with minor physical abnormalities [13]. In one twin, two of 50 metaphases had a 45,XX,-22 karyotype. Significantly, two cells of the 50 metaphases of the second twin had an apparently normal chromosome number, and one cell had 46 chromosomes with a dicentric ring. The remaining cells in both patients were 46,XX,r(22).

Heterozygous contiguous gene deletion at 22q13 or mutations in the *SHANK3* gene (OMIM 606230), located within the minimum critical region, cause Phelan-McDermid syndrome (PHMDS, OMIM 606232). The frequent clinical findings are intrauterine and postnatal growth retardation, intellectual disability, speech delay, delayed motor development, microcephaly, large and misshapen ears, mild hypertelorism, strabismus, epicanthic folds, ptosis of the upper lips, bushy eyebrows and synophrys, a depressed and broad nasal bridge, short mandible, malocclusion and irregular position of the teeth, high palate, clinodactyly of the little fingers, partial syndactyly between the 2nd and 3rd toes, hypotonia, seizures and behavior problems.

Here, for the first time, we report a patient with a compound phenotype who exhibits ring chromosome 22, mosaic monosomy for chromosome 22 in lymphocytes and skin fibroblasts, and a microdeletion at 3q13.31 of maternal origin. Both microdeletions, del3q13.31 and del22q13.32-q13.33, were also detected by real-time PCR in the buccal epithelium.

The single-gene deletion of *TUSC7* at 3q13.31 in the index patient is located in the region of the 3q13.31 deletion syndrome (OMIM 615433). Although the *TUSC7* gene encodes the long non-coding RNA associated with various types of cancer [14], its expression has been shown to be closely related to the expression of the *LSAMP* gene [15], which is involved in neurodevelopmental impairments [16, 17].

### Clinical report

**Fig. 1:**
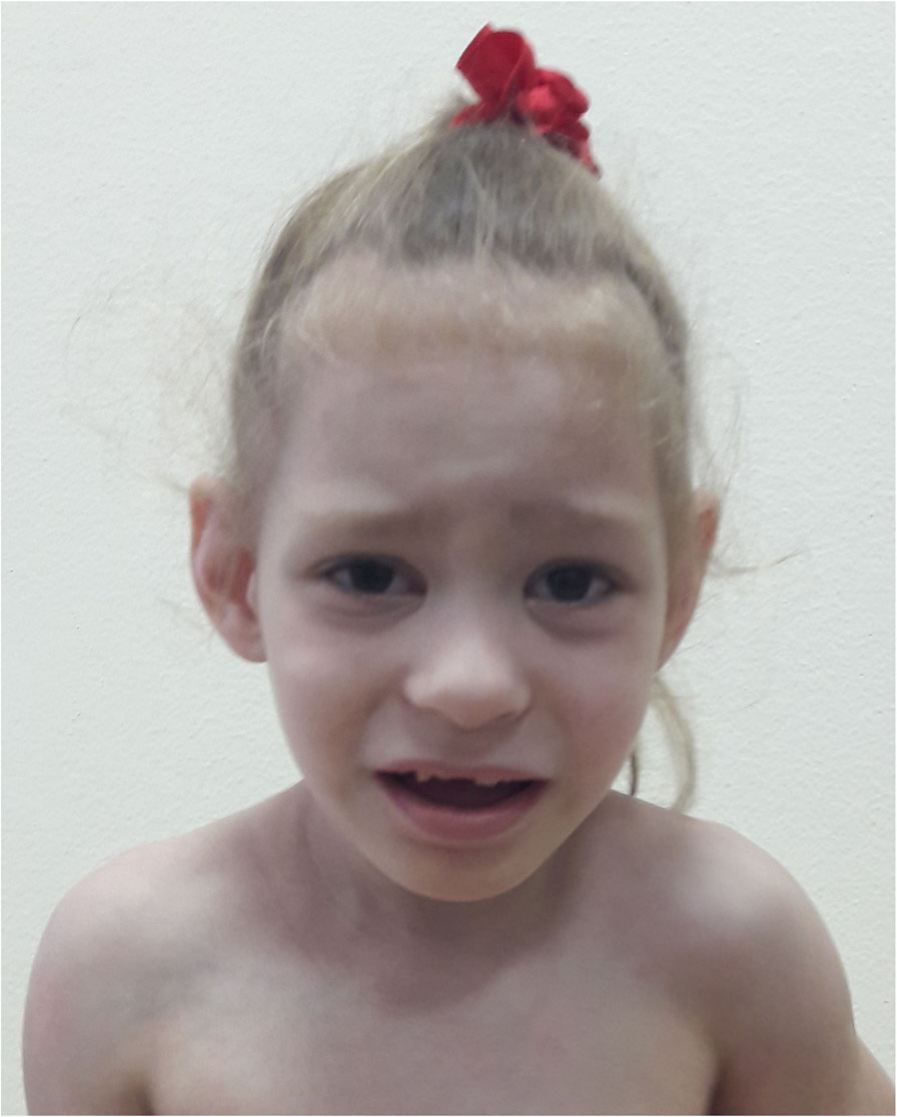
The patient at 4 years of age (note plagiocephaly, high anterior hairline, broad forehead, epicanthus, widely spaced eyes, upslanted palpebral fissure, upper eyelid fullness, straight eyebrows, prominent ears, wide and depressed nasal bridge, bulbous nose, smooth and short philtrum, and thin vermillion of the upper lip)

The patient (Fig. 1), a 4-year-old girl, was referred to the clinical geneticist for the first time when she was one year and eight months old because of development delay, hyperexcitability, mood swings, and sleep disturbance. The girl did not walk alone, did not speak, and her head circumference did not increase. She is an only child of non-consanguineous healthy parents. Her father’s nephew is intellectually disabled and receives education at home.

The patient was born at the 34^th^ week of gestation via Cesarean section. Her birth weight was 1422 g (< 3^rd^ centile); birth length was 48 cm (25^th^ centile); head circumference was 25 cm (< 3^rd^ centile); and chest circumference was 24 cm (< 3^rd^ centile). Her Apgar score was 6. She was able to sit at the age of 8 months and walk independently at the age of 2 years.

The patient had neonatal hypotonia and hyporeflexia. Anticonvulsant therapy was prescribed at the age of 2 months because of seizures. At the age of 3 months, the seizures stopped, and the drugs were discontinued. After birth, the girl was placed on artificial feeding and had trouble gaining weight.

Currently, the patient is regularly observed by a neurologist, with diagnoses of cerebral palsy, atonic-astatic form, psychomotor and speech development delay, and by a psychiatrist due to mental disorder and decreased intelligence to the level of pronounced debility, psychomotor disinhibition syndrome, and unformed control functions for physiological sentiments.

At the age of 4 years, the girl’s weight was 14.5 kg (25^th^ centile); height was 108 cm (95^th^ centile); and head circumference was 45.5 cm (<2^th^ centile). She exhibited plagiocephaly, flat occiput, high anterior hairline, broad forehead, micrognathia, epicanthus, widely spaced eyes, upslanted palpebral fissure, upper eyelid fullness, straight eyebrows, prominent ears, wide and depressed nasal bridge, bulbous nose, smooth and short philtrum, thin vermillion of the upper lip, widely spaced teeth, high palate, clinodactyly (radial, F5, bilateral), proximally placed thumb (bilateral), thickening of the distal phalanx of the thumb (bilateral), pes planus, sandal gap, cutaneous syndactyly of the toes (T2-3, bilateral), short toes (T3-5, bilateral), dysplastic nails of the feet, asthenic body, wide umbilical ring, and a sacral dimple. The patient demonstrated aggression, signs of autism, attention deficit hyperactivity disorder (ADHD), and enuresis.

When the girl was 6 months old, echoencephalography showed ventriculomegaly. At the age of 1 year and 6 months, magnetic resonance imaging of the brain revealed a Dandy-Walker variant: hypoplasia of the cerebellar tonsils. Echocardiography revealed small heart anomalies, including an open oval window with a minimal left-right discharge, an enlarged coronary sinus, and a diagonal false chord of the left ventricle.

## Methods

The patient was subject to aCGH analysis due to her compound phenotype and r(22), as determined by conventional cytogenetic analysis of her blood lymphocytes. The aCGH findings were confirmed by real-time PCR in three different tissues: lymphocytes, skin fibroblasts, and buccal epithelium. FISH analysis was used to confirm the ring form of chromosome 22 and to determine the level of cells with monosomy 22 among lymphocytes and skin fibroblasts. SNP analyses identified the maternal origin of the ring chromosome. All techniques were performed by using equipment of the Center "Medical Genomics" of Research Institute of Medical Genetics, Tomsk NRMC.

### Materials

For molecular genetic analyses, peripheral blood was collected in tubes containing EDTA. A primary culture of the patient’s skin fibroblasts was obtained from two full-thickness skin biopsies. A buccal smear was collected with a cotton swab, which was transported in a dry tube for further DNA extraction.

### Cytogenetic analyses

Conventional cytogenetic analysis was performed based on GTG-banded metaphases from peripheral blood lymphocytes from the patient at a 400-band resolution.

### Fibroblast culture

The biopsies of fibroblasts were washed twice with Hank’s solution containing antibiotics and antifungal agents and then treated with 0.2% collagenase in culture medium for 3 h at 37°C. The suspension was subsequently cultured in AminoMax culture medium. A confluent monolayer formed in one day.

### Array-based Comparative Genomic Hybridization (aCGH) analyses

aCGH was performed using the SurePrint G3 Human CGH Microarray Kit (8×60K) (Agilent Technologies, Santa Clara, CA USA), according to the manufacturer’s recommendations. Labeling and hybridization of patient and reference DNA (#5190-3796, Human Reference DNA, Agilent Technologies, Santa Clara, CA USA) were performed using enzymatic labeling and hybridization protocols (v. 7.5 Agilent Technologies, Santa Clara, CA USA). Array images were acquired with an Agilent SureScan Microarray Scanner (Agilent Technologies, Santa Clara, CA USA). Data analysis was performed using Cytogenomics Software (v. 3.0.6.6) (Agilent Technologies), the publicly available Database of Genomic Variants (DGV), and the Database of Chromosomal Imbalance and Phenotype in Humans employing Ensembl Resources (DECIPHER). Annotations of the genes located within the region of genomic imbalance were retrieved from the NCBI Gene Database, OMIM, and the literature.

### Confirmation of copy number variation using quantitative real-time PCR

Target sequences within and outside the deleted chromosomal regions and specific amplification primers for quantitative real-time PCR assays were selected using Primer 3 software (Additional file 1). The presence of 3q13.31 microdeletion, 22q13.32 microduplication and 22q13.32-q13.33 microdeletion was tested using genomic DNA from peripheral blood lymphocytes from the patient, her parents, and maternal mother (maternal father was not available for the analysis) as well as in cultured skin fibroblasts and a buccal smear from the patient and using the AriaMX Real-Time PCR System (Agilent Technologies, Santa Clara, CA USA). Control genomic DNA was obtained from the peripheral blood lymphocytes, skin fibroblasts and a buccal smear of a healthy donor. The control gene was *HEXB*, which encodes the beta subunit of hexosaminidase and is located at 5q13 (Additional file 1).

### Fluorescent *In Situ* Hybridization (FISH)

FISH was performed using PCR-based probes for the centromeres of chromosomes 14 and 22 and the *TBC1D22A* gene located close to del22q13.32-q13.33 in lymphocytes and cultured skin fibroblasts from the proband following the standard protocol. *E. coli* clones carrying plasmids with inserts of centromere-specific alpha-satellite DNA sequences as well as the BAC clone RP11-569D9 were kindly provided by Professor M. Rocchi (Resources for Molecular Cytogenetics, Institute of Genetics, Bari, Italy). The probe for the *TBC1D22A* gene was generated using a long-range PCR kit (BioLabMix, Novosibirsk, Russia) (Additional file 1). Probes 14/22 and TBC1D22A were labeled with TAMRA-dUTP and Fluorescein-dUTP (BioSan, Russia), respectively.

The results of the aCGH and FISH analyses are described further according to the International System for Human Cytogenomic Nomenclature (ISCN 2016).

### SNP analyses

Nine SNPs (rs11541025, rs2272837, rs8238, rs11703226, rs6010218, rs28637964, rs11547734, rs28379706, rs5771206) located at 22q13.32-q13.33 were selected to determine the parental origin of chromosome 22 with deletion. SNP investigation was performed via SNaPshot analysis.

## Results

**Fig. 2:**
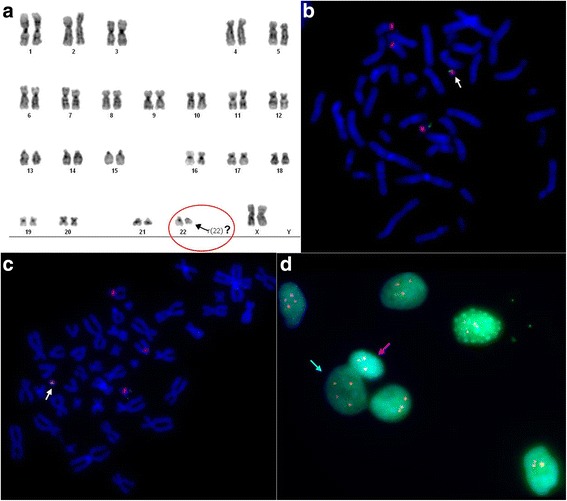
Cytogenetic analysis. **a** Conventional cytogenetics analysis: ring chromosome 22; **b** FISH analysis with centromere-specific probes for chromosomes 14/22 (red) and a PCR-based probe for TBC1D22A (green) in cultured lymphocytes from the proband. The arrow indicates ring chromosome 22. C, D - FISH analysis with centromere-specific probes for chromosomes 14/22 (red) and PCR-based probes for TBC1D22A (green) in cultured skin fibroblasts from the proband. Ring chromosome 22 is designated by a white arrow (**c**); blue and pink arrows designate 46,XX,r(22) and 45,XX,-22 cells, respectively (**d**)

**Fig. 3:**
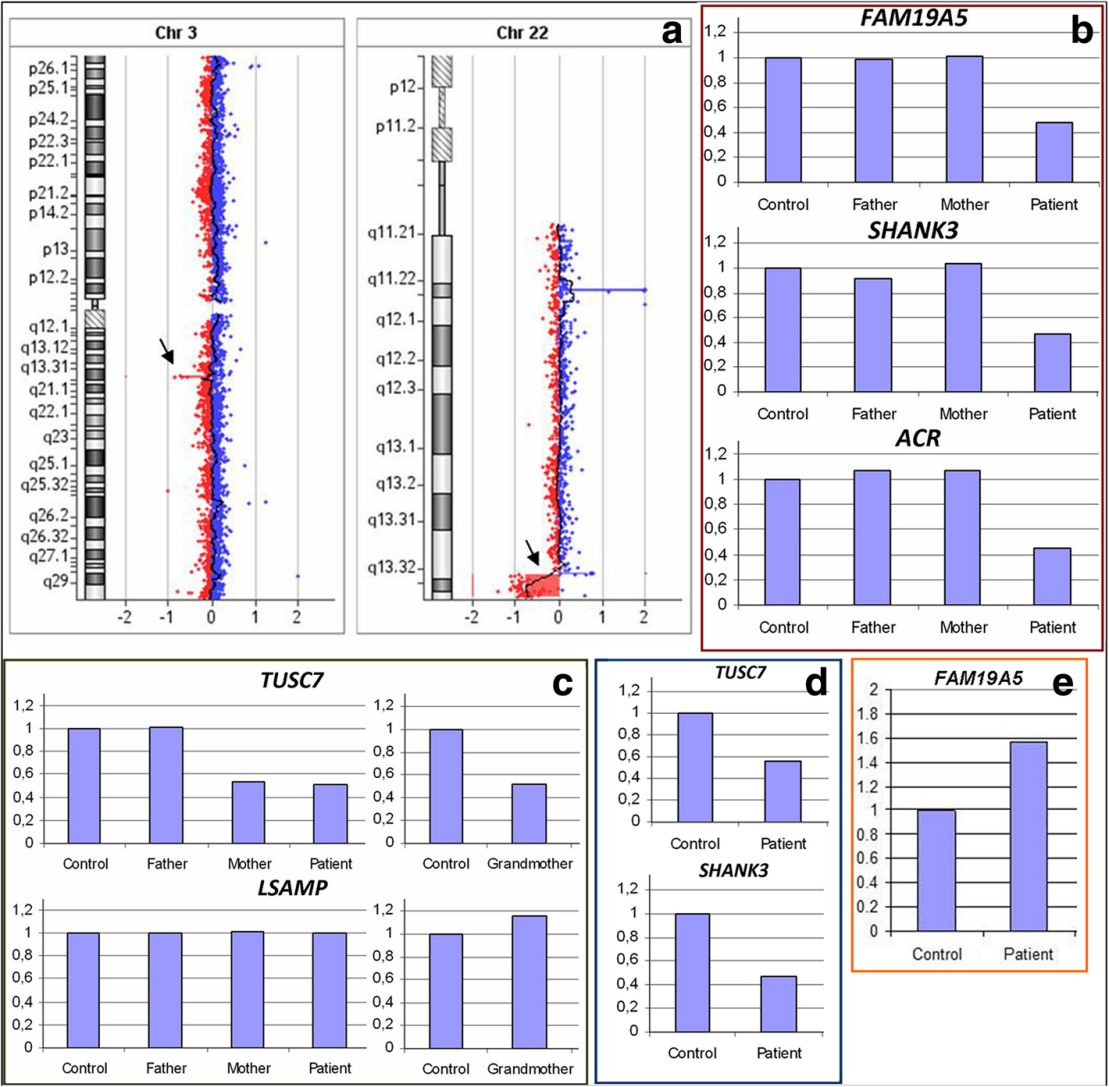
Molecular genetic analysis. **a** An aCGH image of chromosomes 3 and 22 in the lymphocytes of the patient. Deletions are designated by arrows. **b** Confirmation of the deletion at 22q13.32-q13.33 by quantitative real-time PCR analysis. **c** Confirmation of the deletion at 3q13.31 by quantitative real-time PCR analysis. **d** Identification of the deletions at 3q13.31 and 22q13.32-q13.33 in the buccal epithelium by quantitative real-time PCR analysis. **e** Confirmation of the duplication at 22q13.32 by quantitative real-time PCR analysis

Metaphase analysis of the G-banded chromosomes from peripheral blood lymphocytes showed a karyotype 46,XX,r(22) [11] (Fig. 2a). FISH analysis detected 8% of monosomic cells (Table 1). aCGH using an Agilent 60K microarray revealed del3q13.31, dup22q13.32, and del22q13.32-q13.33 of 382 kb, 180 kb, and 2.024 Mb, in size, respectively: arr[hg19] 3q13.31(116233164_116615500)×1,22q13.32(48886812_49059015)×3, 22q13.32-q13.33(49115584_51178264)×1 (Fig. 3a). The distal breakpoint of dup22q13.32 is located within intron 2 of the *FAM19A5* gene, i.e., exons 1-2 are duplicated, and the proximal breakpoint of del22q13.32-q13.33 is located within intron 3 of the *FAM19A5* gene, i.e., exon 4 is deleted. The 3q13.31 microdeletion involved the single *TUSC7* gene. The microdeletion was confirmed via quantitative real-time PCR analysis and was shown to be inherited from the intellectually normal mother and grandmother (Fig. 3c). *TUSC7* is known to be regulated together with the neighboring *LSAMP* [15], which is associated with neuropsychiatric disorders in patients with 3q13.31 deletion syndrome (OMIM615433). We also investigated the number of copies of the latter gene, which appeared to be normal (Fig. 3c). Similar microdeletions and reciprocal microduplications were present in DGV in one and two reports, respectively, and in DECIPHER (nos. 264508 and 299972). However, the gene located within this region in DECIPHER is *LSAMP,* with the coordinates - chr3:115521235-117716095 (hg19) vs. chr3:115521210-116164385 (hg19) in DGV. The deletion in the index patient (chr3:116233164-116615500 (hg19)) is within the DECIPHER region.

**Table 1:** Cytogenetic results for lymphocytes and skin fibroblasts from the index patient

Cell type/duration of culturing or passage number	FISH				
	D14Z1/D22Z1×3	D14Z1/D22Z1×4		Total	% of cells with monosomy 22
Lymphocytes/72 h	41	438		479	8.56
Fibroblasts/P1	27	86		113	23.89
	Metaphase analysis				
Cell type/duration of culturing	45,XX,-22	46,XX,r(22)	46,XX,+mar	Total	% of cells with monosomy 22
Lymphocytes/72 h	-	11	-	11	0

Footnotes. D14Z1/D22Z1×3 – monosomy 14 or 22; D14Z1/D22Z1×4 – two chromosomes 14 and 22; P1 – first passage

The 22q13.32-q13.33 microdeletion overlaps with the region of PHMDS and includes 43 RefSeq genes. The main candidate gene within this region is *SHANK3* (606230) [18]. Real-time PCR confirmed the presence of the microdeletion with primers for the *FAM19A5, SHANK3*, and *ACR* genes (Fig. 3b). According to the SNP analysis, the 22q13.32-q13.33 microdeletion originated *de novo* on the maternal chromosome.

The ring chromosome 22 was confirmed via FISH using centromere-specific DNA probes for chromosomes 14 and 22 and for the *TBC1D22A* gene located in the intact part of chromosome 22 close to del22q13.32-q13.33 (Fig. 2b). In addition, 8% of lymphocytes were observed to exhibit monosomy for chromosome 22 (nuc ish(D14Z1/D22Z1×3)[41/479],(TBC1D22A×1)[41/479]).

Skin fibroblasts of the patient were investigated using the Agilent 60K microarray as well. The 22q13.32-q13.33 microdeletion (arr[hg19]22q13.32-q13.33(49084185_51043490)×1) was found and further confirmed via real-time PCR, while the 3q13.31 deletion and 22q13.32 duplication were not revealed by aCGH but were also determined by real-time PCR (Fig. 3e). aCGH demonstrated the shift of chromosomal profiles at 3q13.31 and 22q13.32 towards the deletion and duplication, respectively, which, however, did not reach the significance level due to the high variance of the fluorescence intensity over the chromosomes. FISH analysis showed that 24% of the fibroblasts initially had monosomy of chromosome 22 (Fig. 2c and d): nuc ish(D14Z1/D22Z1×3)[27/113], (TBC1D22A×1)[27/113].

The buccal epithelium was investigated via real-time PCR. Both 3q13.31 and 22q13.32-q13.33 deletions were confirmed (Fig. 3d).

## Discussion

We present a patient with a compound phenotype and a combination of chromosomal abnormalities: del3q13.31 (single *TUSC7* gene), dup22q13.32 (LOC284933 and exons 1-2 of *FAM19A5* gene), ring chromosome 22 associated with del22q13.32-q13.33 (43 RefSeq genes), and mosaic chromosome 22 monosomy in 8% of lymphocytes and 24% of fibroblasts. Both microdeletions were present in lymphocytes, fibroblasts, and the buccal epithelium. The microduplication was present in lymphocytes and fibroblasts; the buccal epithelium was not investigated. The del3q13.31 was inherited from the apparently healthy mother and grandmother and is located within the region of the 3q13.31 deletion syndrome (OMIM 615433).

The only gene located within the deleted 3q13.31 region is *TUSC7*, also called LSAMP antisense RNA3. It is a putative suppressor gene in various tumors, including glioma [19]. Low *TUSC7* expression is associated with significantly unfavorable survival and may be a risk factor for distant metastases [20]. Frequent deletions in the 3q13.31, including *LSAMP* and *TUSC7*, have also been identified in patients with osteosarcomas [14]. Although the copy number of the *LSAMP* gene is preserved in our patient, *LSAMP* and *TUSC7* expression are known to be closely related [15]. The authors also showed that, although the patient with acute myeloid leukemia had a 250-kb deletion in 3q13.31, which included the *TUSC7* gene but not *LSAMP*, the expression of both genes was increased by an unknown mechanism. *LSAMP*, implicated in the regulation of emotional and social behavior in mice [21] and associated with major depressive disorder and schizophrenia in humans [16, 17], is also one of the candidate genes for the 3q13.31 deletion syndrome.

Del22q13.32-q13.33 in our patient originated *de novo* on the maternal chromosome. This deletion is associated with the origin of the ring chromosome and includes part of the minimal critical region of PHMDS ([hg19] 22q13.33(51045516_51187844)) with the *MAPK8IP2, ARSA, SHANK3*, and *ACR* genes. PHMDS is a developmental disorder (OMIM 606232). The symptoms of the index proband commonly associated with PHMDS are listed in Table 2.

**Table 2:** Symptoms in the index patient common to Phelan-McDermid syndrome and mosaic monosomy for chromosome 22 as well as atypical symptoms

Index patient	Phelan-McDermid syndrome	Mosaic monosomy 22
Developmental delay	+	+
Sleep disturbance	+	
Neonatal hypotonia^a^		+
Seizures	+	
Speech delay	+	
Low weight^a^		+
Accelerated growth	+	
Microcephaly	+	+
**Plagiocephaly**		
**Flat occiput**		
**High anterior hairline**		
Broad forehead^a^		+
Micrognathia^a^		+
Epicanthus	+	+
Widely spaced eyes^a^		+
**Upslanted palpebral fissure**		
**Upper eyelid fullness**		
**Straight eyebrows**		
Prominent ears	+	+
Wide and depressed nasal bridge^a^		+
Bulbous nose	+	
Smooth and short philtrum	+	
Thin vermillion of the upper lip^a^		+
Widely spaced teeth	+	
High palate^a^		+
Clinodactyly (radial, F5, bilateral)^a^		+
**Proximally placed thumb (bilateral)**		
**Thickening of distal phalanx of thumb (bilateral)**		
**Pes planus**		
**Sandal gap**		
Cutaneous syndactyly of the toes (T2-3, bilateral)^a^		+
**Short toes (T3-5, bilateral)**		
Dysplastic nails of the feet	+	
**Asthenic body**		
**Wide umbilical ring**		
Sacral dimple	+	
Aggression	+	
Signs of autism	+	
Attention deficit hyperactivity disorder	+	
**Enuresis**		
Dandy-Walker variant^b^	+	
Ventriculomegaly	+	
Small heart anomalies^a^		+

Footnotes. Symptoms of the index proband never before described in patients with Phelan-McDermid syndrome or mosaic monosomy for chromosome 22 are shown in bold. ^a^-symptoms specific to monosomy 22. ^b^ - patient P60 from [37] had Dandy-Walker malformation.

The most important contributing factor to PHMDS is *SHANK3* haploinsufficiency [22]. Mutations in this gene have also been associated with schizophrenia [23]. Analysis of human neurons differentiated from the induced pluripotent cells of patients with PHMDS showed impaired synaptic transmission and increased input resistance [24].

The instability of r(22) in the index patient led to the loss of chromosome 22 in a portion of cells, resulting in monosomy for chromosome 22. Non-mosaic monosomy for chromosome 22 is incompatible with life. To date, only seven cases of monosomy 22 have been published [25–31], four of which were mosaic. The oldest described child was three years old. None of the patients had r(22), which could somehow, during the early stages of the development before its elimination from the cell, compensate for the phenotype. The symptoms of our proband are similar to those described in patients with mosaicism for monosomy 22 and are listed in Table 2. In addition, the index patient exhibits some symptoms that have not been described in any patient with either of the anomalies discussed above.

How can the clinical phenotype of our patient be interpreted? To our knowledge, two explanations may exist: phenotypic variability and a combined effect of del22q13.32-q13.33 and mosaic monosomy for chromosome 22. Phenotypic variability is typical for the manifestation of most CNV-associated syndromes. Variability can be associated with the variability of the breakpoints. In addition, the phenotype of a patient may also result from a combination of genetic and non-genetic modifiers.

The heterogeneous clinical phenotype in carriers of the ring chromosome 22 was first explained by mosaicism with monosomy 22 by Lejeune in 1968 [32]. Later, Stoll and Roth described a family with 46,XX/46,XX,r(22) and 46,XY/46,XY,r(22) karyotypes in three generations of individuals with both normal development and severe mental retardation. In these individuals, 40-50% of their lymphocytes had a ring chromosome 22, and a few cells were observed to carry two ring chromosomes. Skin biopsies were refused [33]. In 2014, a patient with a developmental delay and dysmorphic features was described, from whom 12% of lymphocytes and 40-48% of fibroblasts had r(22), and the remaining cells had a normal karyotype - 46,XX/46,XX,r(22). In some metaphase cells of a patient, the ring chromosome was duplicated or lost [23]. In our patient, the initial metaphase analysis revealed only cells with the 46,XX,r(22) karyotype. Using FISH, we performed a search for mosaicism of the ring chromosome 22 in interphase nuclei of peripheral blood lymphocytes and skin fibroblasts of the index patient. Approximately 8% of lymphocytes and 24% of fibroblasts had monosomy 22 in combination with 46,XX,r(22) cells. The discrepancy between the results of these two analyses can be due to the low level of monosomic cells among lymphocytes detected by FISH, which was not revealed by metaphase analysis, as well as to the potential inability of monosomic cells to proliferate in vitro.

To the best of our knowledge, only four live-born infants with mosaicism for monosomy of chromosome 22 have been described. The first one is a 2-year-old male child with moderate psychomotor retardation, generalized hypotonia, large ears, epicanthus, synophrys, and cutaneous syndactyly between all the fingers [28]. The patient’s karyotype was 45,XY,-22/46,XY [12/50], i.e., 24% of lymphocytes were monosomic. The second case was published by Verloes et al. [31], who observed a slightly dysmorphic and mentally defective three-year-old child with a 45,XY,-22/46,XY karyotype. Chromosome 22 was absent in 10.5% of lymphocytes and 8.3% of fibroblasts. The third case was a newborn with gastroschisis and absent cerebral diastolic flow. The baby died on its second day of life. The karyotype was 45,XY,-22/46,XY [3/50] [26]. The fourth child was an abnormal premature male infant with a 45,XY,-22/46,XY [15/100] karyotype [29]. He had a unique facial appearance, similar to those with DiGeorge syndrome (OMIM 188400), hypertonicity, limitation of extension at the major joints, and flexion contractures of all fingers. However, the direct comparison of the phenotypes of these patients with the symptoms of our proband seem to be incorrect because, in addition to monosomy for chromosome 22, she also has a deletion at 22q13.32-q13.33.

For the index patient, among 43 identified symptoms, 18 have previously been described in other patients with PHMDS and 15 in patients with mosaic monosomy 22, while 14 symptoms have never been observed in any of the mentioned cases (Table 2). These symptoms include skeletal abnormalities, facial dysmorphism, wide umbilical ring, and enuresis. Significantly, two important papers concerning the *FAM19A5* gene, which is disrupted by the CNV breakpoints in the index patient, have been recently published. The first paper demonstrates that the gene is a crucial candidate for modulating osteoclast formation and bone disorders [34], i.e., we can assume that the skeletal abnormalities are due to FAM19A5 protein deficiency. In addition, the second paper is the first to show that FAM19A5 is highly expressed and secreted by adipocytes and adipose tissue protein [35]. Thus, its deficiency can probably be related to the low weight and asthenic body of the proband. Moreover, *FAM19A5* is also expressed in the brain and acts as a modulator of immune response in nervous cells, which can make it responsible for neuropsychiatric processes [36].

## Conclusions

In conclusion, we present the first patient with a compound phenotype and a combination of a 382-kb deletion at 3q13.31, encompassing the single *TUSC7* gene, a 180-kb duplication at 22q13.32, a 2.024-Mb deletion at 22q11.32-q11.33, including part of the PHMDS critical region, and monosomy for chromosome 22 in 8% of lymphocytes and 24% of fibroblasts. The presence of deletions was detected by real-time PCR in the buccal epithelium. The clinical significance of the 3q13.31 deletion is unclear, though the *TUSC7* gene is associated with various types of tumors. Special attention should thus be paid to the early prevention and diagnosis of cancer in a carrier. In addition, only the combination of the 22q13.32-q13.33 deletion and monosomy for chromosome 22 allowed us to explain most of the clinical features of the patient. Therefore, in cases with a compound phenotype, it is important to use a combination of different methods and sometimes to investigate more than one tissue. The presence of monosomic cells in patients with a ring chromosome indicates ring chromosome instability. A monosomic karyotype can be the intermediate step in the process of chromosomal defect correction, which can underlie the chromosomal therapy of genetic diseases.

## Additional file

Additional file 1: Primers for real-time PCR and FISH-probe synthesis. (DOC 41 kb)

## Abbreviations

aCGH: Array comparative genomic hybridization
CNV: Copy number variation
DECIPHER: Database of Chromosomal Imbalance and Phenotype in Humans Using Ensembl Resources
iPSC: Induced pluripotent stem cell
OMIM: Online Mendelian Inheritance in Man
PCR: Polymerase chain reaction
PHMDS: Phelan-McDermid syndrome
SNP: Single-nucleotide polymorphism
