# Supplementary material for: Compound phenotype in a girl with r(22), concomitant microdeletion 22q13.32-q13.33 and mosaic monosomy 22
Source: Mol Cytogenet. 2018 Apr 27;11:26. doi: 10.1186/s13039-018-0375-3 (PMC5923029; doi:10.1186/s13039-018-0375-3)
Supplement: Supplementary file 1 — Primers for real-time PCR and FISH-probe synthesis. (DOC 41 kb) [file 13039_2018_375_MOESM1_ESM.doc]

Additional file 1

Primers for real-time PCR and FISH-probe synthesis

| Region | Primer | Sequence | Analysis |
| --- | --- | --- | --- |
| 3q13.31 | LSAMP F | 5’- GAGATCCTTGGCATCACCAG-3’ | Real-time PCR |
|  | LSAMP R | 5’- TCACAGTGACCTTGACTTGTTTG-3’ |  |
|  | TUSC7 F | 5’-GAGCCAGCTTCACTGGAAAC-3’ |  |
|  | TUSC7 R | 5’-CCCTGTGGCTCTACAAGAGG-3’ |  |
| 5q13.3 | HEXB F | 5’-CCGGGCACAATAGTTGAAGT-3’ |  |
|  | HEXB R | 5’-TCCTCCAATCTTGTCCATAGC-3’ |  |
| 22q13.32 | FAM19A5ex2 F | 5’-GGCACCTGTGAGATTGTGAC-3’ |  |
|  | FAM19A5ex2 R | 5’-CGATCTGCCCCTTTCTACAC-3’ |  |
| 22q13.32-q13.33 | FAM19A5ex4 F | 5’-TGAGGTTGGGTTTGTCATCA-3’ |  |
|  | FAM19A5ex4 R | 5’-CTGCCCTCGAAGGTGTCTAC-3’ |  |
|  | SHANK3ex2 F | 5’-CAGGACGCGCTCAACTATG-3’ |  |
|  | SHANK3ex2 R | 5’-GGTTGGGCGGGTACTCCT-3’ |  |
|  | ACRex2 F | 5’-GGTTACGGTTCAGGCAAAAC-3’ |  |
|  | ACRex2 R | 5’-AGTGAGCACCCATCGTGAAT-3’ |  |
| 22q13.31 | TBC1D22A F1 | 5’-TTGTGGTGGAGCACCTGTTT-3’ | FISH |
|  | TBC1D22A R1 | 5’-AAGCAGCCAATGACCCTCTC-3’ |  |
|  | TBC1D22A F2 | 5’-TGACCACCATCCTCATGCAC-3’ |  |
|  | TBC1D22A R2 | 5’-AGATTCAGCAGACGCACCAA-3’ |  |
|  | TBC1D22A F3 | 5’-CAATCCTCCCGTGCTATCCC-3’ |  |
|  | TBC1D22A R3 | 5’-AGGCGAGATGACGCATAAGG-3’ |  |
|  | TBC1D22A F4 | 5’-CCTTTGTGGGATGGGATGCT-3’ |  |
|  | TBC1D22A R4 | 5’-GCGCCTCCCTTAGTGATACC-3’ |  |
